# Anti-Tumor Effects of Metformin in Animal Models of Hepatocellular Carcinoma: A Systematic Review and Meta-Analysis

**DOI:** 10.1371/journal.pone.0127967

**Published:** 2015-06-01

**Authors:** Juan Li, Pratika Y. Hernanda, Wichor M. Bramer, Maikel P. Peppelenbosch, Judith van Luijk, Qiuwei Pan

**Affiliations:** 1 Department of Gastroenterology and Hepatology, Erasmus MC Cancer Institute, Erasmus University Medical Center, Rotterdam, The Netherlands; 2 Laboratory of Medical Genetics, Biomolecular Research Centre, Wijaya Kusuma University, Surabaya, Indonesia; 3 Medical Library, Erasmus University Medical Center, Rotterdam, The Netherlands; 4 SYRCLE at Central Animal Laboratory, Radboud University Medical Centre, Nijmegen, The Netherlands; Taipei Veterans General Hosptial, TAIWAN

## Abstract

**Background:**

Several studies have reported that metformin can reduce the risk of hepatocellular carcinoma (HCC) in diabetes patients. However, the direct anti-HCC effects of metformin have hardly been studied in patients, but have been extensively investigated in animal models of HCC. We therefore performed a systematic review and meta-analysis of animal studies evaluating the effects of metformin on HCC.

**Methods:**

We collected the relevant studies by searching EMBASE, Medline (OvidSP), Web of Science, Scopus, PubMed Publisher, and Google Scholar. Studies were included according to the following inclusion criteria: HCC, animal study, and metformin intervention. Study quality was assessed using SYRCLE’s risk of bias tool. A meta-analysis was performed for the outcome measures: tumor growth (tumor volume, weight and size), tumor number and incidence.

**Results:**

The search resulted in 573 references, of which 13 could be included in the review and 12 included in the meta-analysis. The study characteristics of the included studies varied considerably. Two studies used rats, while the others used mice. Only one study used female animals, nine used male, and three studies didn’t mention the gender of animals in their experiments. The quality of the included studies was low to moderate based on the assessment of their risk of bias. The meta-analysis showed that metformin significantly inhibited the growth of HCC tumour (SMD -2.20[-2.96,-1.43]; n=16), but no significant effect on the number of tumors (SMD-1.05[-2.13,0.03]; n=5) or the incidence of HCC was observed (RR 0.62[0.33,1.16]; n=6). To investigate the potential sources of significant heterogeneities found in outcome of tumor growth (I^2^=81%), subgroup analyses of scales of growth measures and of types of animal models used were performed.

**Conclusion:**

Metformin appears to have a direct anti-HCC effect in animal models. Although the intrinsic limitations of animal studies, this systematic review could provide an important reference for future preclinical animal trials of good quality and clinical development.

## Introduction

Hepatocellular carcinoma (HCC) is the fifth most prevalent cancer worldwide and the third leading cause of death from cancer. Surgical resection and liver transplantation are the only potentially curative treatment for a small proportion of the patients. However, disease recurrence hampers the ultimate success of the treatment [1]. Sorafenib, an oral multikinase inhibitor, was approved to treat advanced HCC. This however only increases patient survival with approximately 2–3 months [2,3]. Therefore, it is necessary to explore new strategies to improve the management of HCC.

Metformin is an oral drug widely used for treatment of type II diabetes. Interestingly, several studies, including observational studies and some randomized controlled trials (RCT), have reported that metformin can affect the risk of hepatocellular carcinoma (HCC) in diabetic patients [4–6]. Although these studies have suggested a preventive effect of metformin on the risk of HCC in these diabetic patients, there is still lacking of investigation whether metformin has direct anti-tumor effect in HCC patients. Nevertheless, substantial research has been performed in animal models of HCC, although the data are still inconclusive. Meta-analyses on data from animal studies can be used to explain clinical observation and to inform clinical trial design.

To better understand the direct effects of metformin on HCC and to pave the way for further prospective clinical study, we performed a systematic review and meta-analysis of currently available data from HCC animal models treated with metformin.

## Materials and Methods

### Review protocol

A protocol for this systematic review was prepared using SYRCLE’s protocol format (https://www.radboudumc.nl/Research/Organisationofresearch/Departments/cdl/SYRCLE/Pages/Protocols.aspx) [7].

### Literature search

A systematic search (conducted on July 2014, without any restrictions on publication data or language) was conducted in Medline (OvidSP), Embase.com, Web of Science, and Scopus. Additional referenes were retrieved from Google Scholar, and unindexed references from PubMed. The searches were designed and executed by an experience information specialist (WB). The search strategy consisted of two main components: hepatocellular carcinoma, and metformin, and results were limited to animal studies. For each element multiple synonyms were searched in title and/or abstract, and when available thesaurus terms (Mesh for medline and Emtree for embase). The full strategy is available in S1 Table.

### Study selection and inclusion criteria

The selection procedure was performed by two independent reviewers (J.L. and P.H.). The exclusion criteria for the title and abstract screening phase include: 1). not primary study; 2). not animal study; 3). not disease of interest (HCC), 4). not intervention of interest (metformin). The following additional criteria were used for full-text screening: 1). full-text not available; 2). double publication; 3). conference abstracts. In case of disagreement between the reviewers, consensus was reached.

### Study characteristics and data extraction

Data was extracted from the full-text papers of the studies. The following items were extracted: author, year, language, species/strain, description of control group, animal gender, age and weight, number of animals in control and experimental group, the method for the establishment of the animal models, metformin dosage, timing, duration and route of metformin administration, and outcome measures (Table 1).

**Table 1:** Characteristics of the included animal studies.

Study	Language	strain/Species	Experimental group	control group	Gender	Age	Weight	animal number: c/exp	Type of animal model	HCC Number/ animal	Dosage	Timing of metformin	Duration of metformin	Adminstration Route	Outcome measures
Chen 2013[9]	English	BALB/c nude mice	HCC+MET	HCC	F	5–6 week	*	5/5.	subcutaneous xenograft	1/1	200 mg/ml in drinking water	10 days after implanation	30 days	orally	TW
Qu 2012[10]	English	BALB/c nude mice	HCC+MET	HCC+saline	M	3–4 week	14.0–16.0 g	12/12; 12/12	orthotopic xenograft	1/1	125& 250 mg/kg /day	10 days after implanation	30 days	i.p.	TV
Miyoshi 2014[11]	English	BALB/c-nu/nu mice	HCC+MET	HCC+PBS	M	8 weeks	20–25 g	10/10; 10/10	subcutaenous xenograft	1/1	1& 2 mg/body/day	after an identifiable mass > 6 mm	14 days	i.p.	TV; cell cycle regulators; angiogenesis
Bhalla 2012[12]	English	C57BL/6J mice	HCC+MET	HCC	M	2 week	*	7/7.	DEN	1/1	250mg/kg/day	2 weeks after DEN	168 days; 252 days	orally	TS, TN; AMPK activation
Kim 2013[13]	English	HBxTg mice	HCC+MET	HBx Tg mice	M	*	*	20/26	HBx Transgenic	1/1	250mg/kg/day	6 weeks of age	462 days	orally	TN; Hepatic CRBP-1 protein level, Akt
DePeralta 2013**[14]	English	Rat (Wistar)	HCC+MET	DEN induction	M	0 week	*	9/9.	DEN	1/1	250 mg/kg/day	8 weeks of age; 12 weeks of age	70 days; 42 days	orally	TI
Cai 2013[15]	English	BALB/c-nu mice	HCC+MET	HCC+PBS	M	6–8 week	*	10/10.	subcutaenous xenograft	4/1	250 mg/kg/day	1 week after transplantation	49 days	i.p.	TV; cell cycle regulators; p-AMPK
Saito 2013[16]	English	NOD/SCID mice	HCC+MET	HCC	*	*	*	5/5.	subcutaenous xenograft	1/1	250 mg/kg/day	just after the transplantation	56 days	i.p.	TV; ki-67, casp3.
Afzal 2012[21]	English	Wistar albino rat	MET+DENA; DENA+MET	DENA induction	M	Adult	100–125g	6/6.	DENA	1/1	125mg/kg/day	Day 1; Day 7.	*	i.p.	animal weight, SGPT/ALT, SGOT/AST
Tajima 2013[17]	English	C57B1/6 mice	non-NAFLD+MET; NAFLD+MET	HFD-HFD	M	8 week	*	6/4; 17/16;4/7;4/8	HFD	1/1	250 mg/kg/day	30 weeks after HFD	210 days	orally	TS, TN; AMPK/mTOR/S6k
Cheng 2014[18]	English	BALB/c-nu mice	HCC+MET	HCC	M	5 week	*	28/10^#^	subcutaenous xenograft	4/1	30 Ag/g body weight	1 week after transplantation	49 days	i.p.	TW, TI; activity of AMPK; Ki-67
Zheng 2013[19]	English	BALB/C nude mice	HCC+MET	HCC+vehicle	*	*	*	8/8; 8/8.	subcutaenous xenograft^§^	1/1	*	*	49–56 days	*	TV; AMPK activation
Xiong 2012[20]	English	BALB/c nude mice	HCC+MET	HCC+PBS	*	*	size of ~100mm^3^	5/5.	subcutaenous xenograft	1/1	40 & 200mg/kg/day	7 days after transplantation	126 days	*	TV, TW

* = not mentioned;

** = only abstract available;

^#^ = indicate tumor number;

^§^ = include two different xenograft model (HCC-LM3 and SMMC7721); MET = metformin; DEN = diethylnitrosamine (liver-specific carcinogen); FBS = fasting blood glucose; HFD = high-fat diet; TV = tumor volume; TW = tumor weight; TS = tumor size; TI = tumor incidence; TN = tumor number.

The outcome measures including HCC growth, number and incidence were included in the meta-analysis. Mean value, standard deviation (SD) and the number of animals per group were extracted. If relevant data were not available in the text but only presented in graphic form, obtaining the data by measuring the graphs using Universal Desktop Ruler (Universal On-screen Digitizer, AVPSoft.).

### Quality assessment of included studies

The SYRCLE’s Risk of Bias tool was used to assess the risk of bias of all included studies [8]. Two independent investigators (J.L. & P.H.) performed quality assessment of all included studies. Disagreements were resolved by discussion.

### Data synthesis and statistical analysis

For the outcome measures of HCC growth and number, the standardized mean difference (SMD) was used as the effect measure. For the outcome measure of HCC incidence, Risk Ratio (RR) was used.

If studies contained multiple independent groups (e.g. different animal models or different time points), they were treated as separate experiments.

Because of expected heterogeneity, the statistical model of analysis used in this meta-analysis was a random effects model. I^2^ was used as a measure of heterogeneity. In order to explore potential causes of heterogeneity, predefined subgroup analyses were conducted for tumor volume, weight and size.

With assistance of RevMan5.3 (Cochrane Library) software, Forest Plots were established. In addition, sensitivity analyses were conducted as to evaluate whether the findings were robust enough to the decisions made. Visual inspection of funnel plots was used to detect publication bias. Our procedures accorded with PRISMA guidelines for reporting systematic review/meta-analysis (S2 Table).

## Results

### Description of the included studies

**Fig 1:**
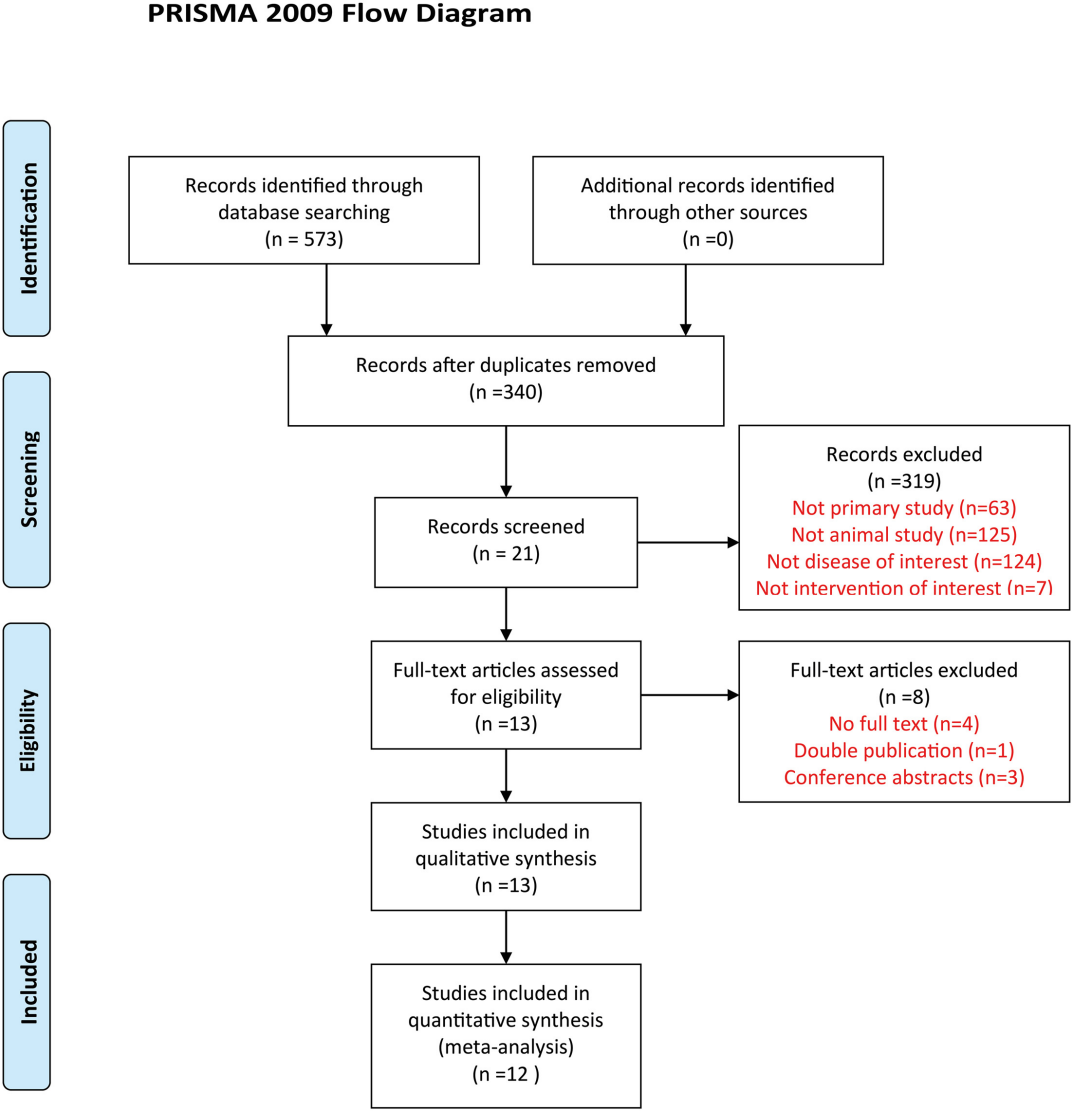
Flow diagram showing literature search and selection results.

The comprehensive search strategy on the effects of metformin on HCC in animal models resulted in 573 records. After duplicates were removed, 340 studies were left. After title and abstract screening, 21 studies were screened full text. Ultimately, 13 studies were included in our systematic review [9–21], of which 12 studies (total 29 animal experiments and 311 animals involved) could be included in the meta-analysis (fig 1) [9–20].

The characteristics of all included studies are described in Table 1. Since the investigation of metformin on HCC using animal models has only been started in recent years, the publication dates of the included studies ranged from 2012 to 2014. Apart from these, the characteristics among these studies varied considerably. The characteristics of animals themselves differed substantially between the studies. Seven of the studies used BALB/c nude mice, and others used C57BL/6J mice, NOD/SCID mice, HBxTg mice, and Wistar rat. Among these studies, seven used a subcutaneous xenograft model, while others used oncogenic compound inducing models. Only half of the studies used the same dosage of metformin (250 mg/kg). Administration timing and duration of metformin varied greatly. Besides, various time-points for outcome measurements were mentioned in the studies.

### Risk of bias and quality of included studies

**Fig 2:**
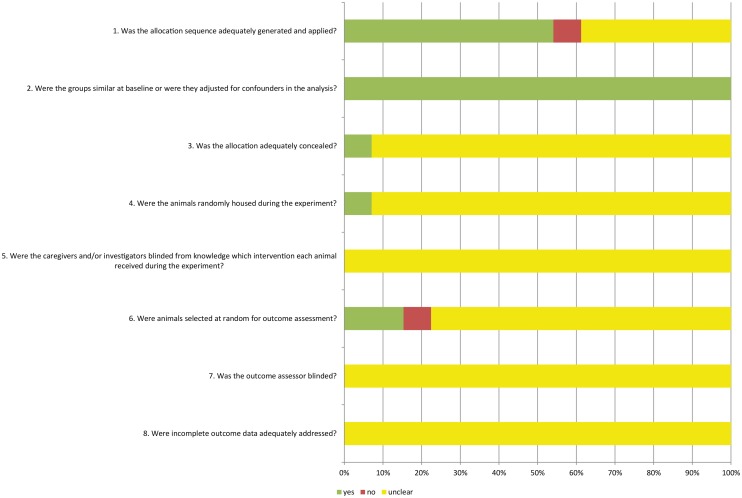
Risk of bias, score (%) per risk of bias item. Yes = low risk of bias, no = high risk bias,? = unclear risk of bias.

Fig 2 shows the results of the risk of bias assessment of the 13 studies included in this systematic review. Based on this assessment, 7 (54%) of the studies stated that the allocation was randomized. Since the background of animals were essentially homogenous, most of the studies didn’t describe the method of randomization. Besides, none of the studies mentioned whether the allocation was adequately concealed. As shown clearly in Fig 2, many items were scored as “unclear”, which indicates that reporting—and presumably experimental design—of these animal studies can be improved.

### Overall analysis of the effects of metformin on HCC growth

**Fig 3:**
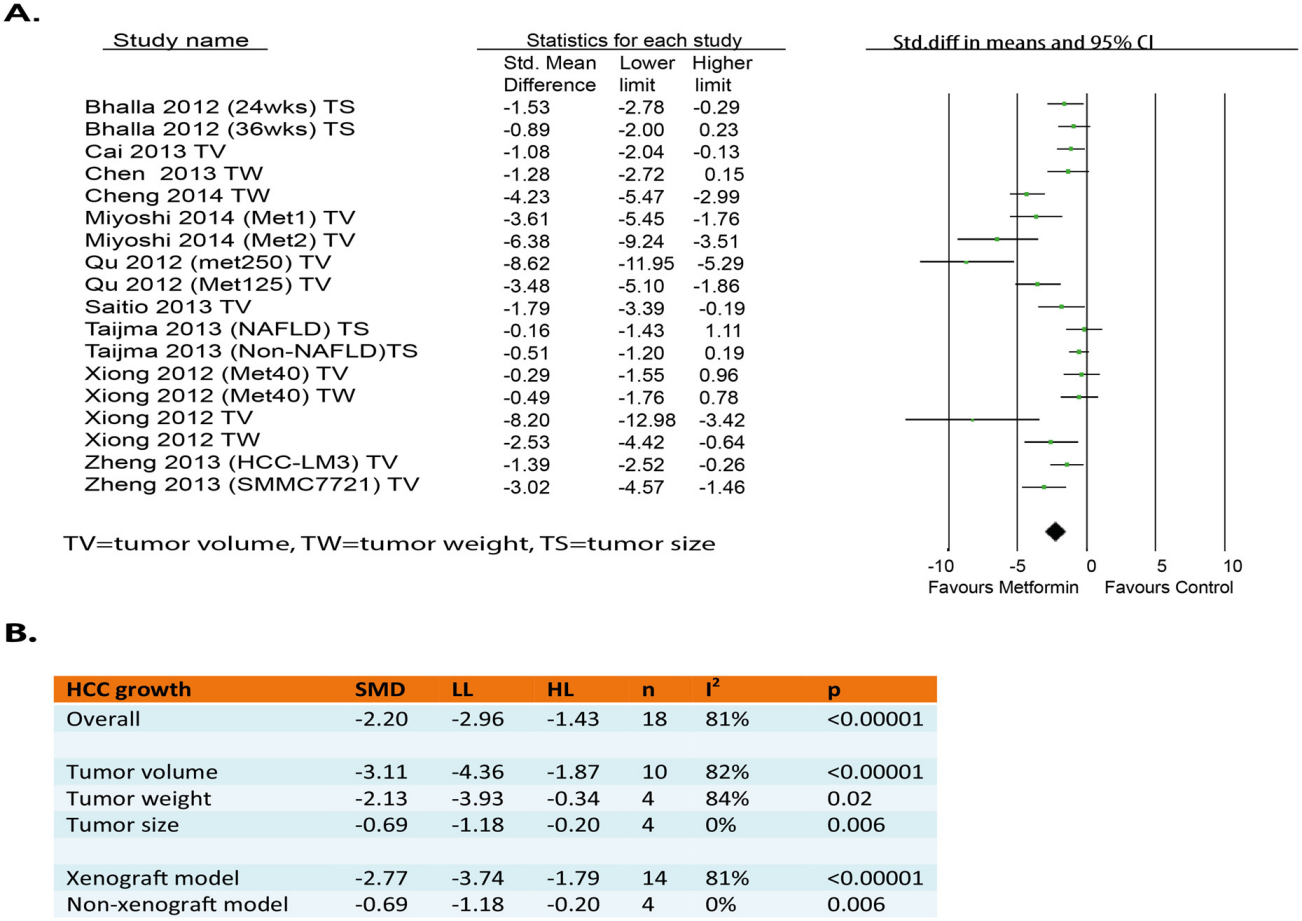
Effects of metformin on tumor growth in HCC animal models. (A) Forest plot and (B) subgroup analysis of the 16 included studies. The forest plot displays the SMD, confidence interval, and effect weight for each study, plus the pooled effect estimate & confidence interval.

Ten out of the twelve studies reported outcomes related to tumor growth (tumor volume, tumor size or tumor weight). These 10 studies contained 18 independent experiments [9–12,15–20]. Of these 18 experiments, 12 showed a significant decrease of tumor growth. None of the experiments showed a significant increase of tumor growth. Meta-analysis of these experiments revealed that metformin intervention had a significant inhibiting effect on HCC growth (SMD-2.20[-2.96, -1.43]; n = 18) (Fig 3A). However, heterogeneity was quite high (I^2^ = 81%).

### Subgroup analysis

To determine whether the effects differed per scale of measurement, the clinically relevant outcome measures including “tumor volume”, “tumor weight” and “tumor size” were analyzed separately in subgroups. As displayed in Fig 3B, even though all three subgroups showed a statistically significant inhibition of growth, the effect on volume (SMD-3.11[-4.36, -1.87]; n = 10) seemed to be larger than on size (SMD-0.69[-1.18, -0.20]; n = 4). Besides, heterogeneity levels significantly decreased in the subgroup analysis of “tumor size”(I^2^ = 0.0%) while still high heterogeneity level were observed in subgroups of “tumor volume”(I^2^ = 82%) and “tumor weight”(I^2^ = 84%).

In addition, a subgroup analysis was performed for the types of HCC model used. This analysis demonstrated that metformin had significant effect on both xenograft (SMD-2.77[-3.74, -1.79]; n = 14) and non-xenograft model (SMD-0.69[-1.18, -0.20]; n = 4), but the former group seemed to be affected by metformin more than the latter group. Subgroup analysis of “non-xenograft model” clearly reduced heterogeneity (I^2^ = 0.0%), while “xenograft model” subgroup analysis did not change high heterogeneity level.

### Effects of metformin on HCC number

**Fig 4:**
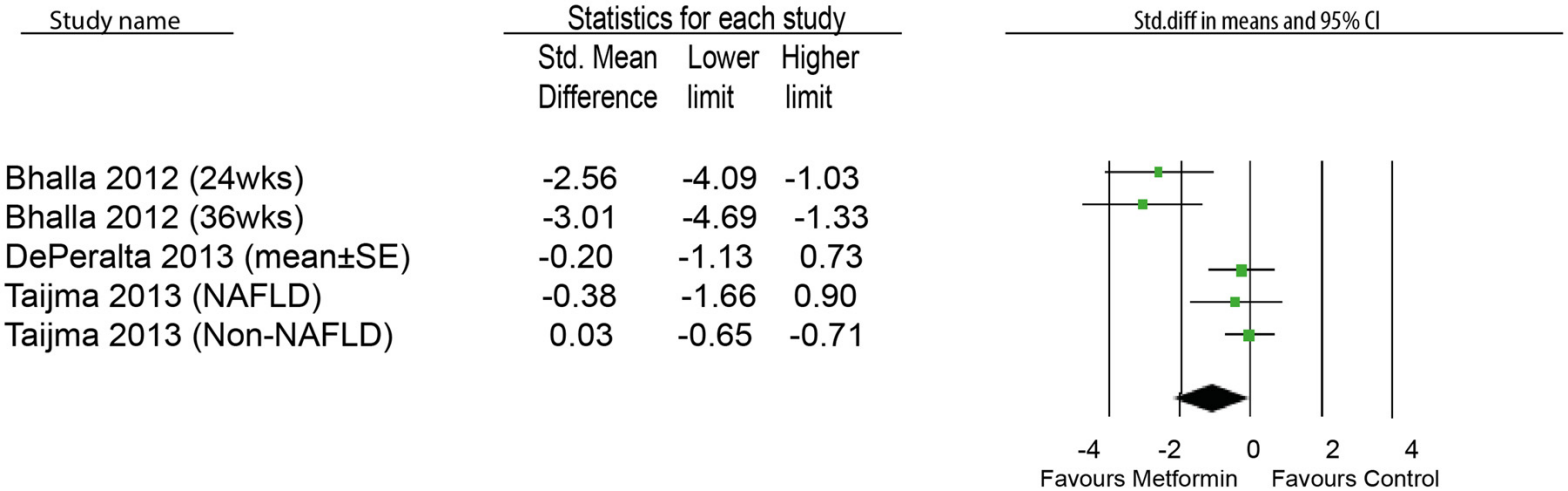
Effects of metformin on tumor number in HCC animal models.

In addition to the analysis of the effect on tumor growth, we also did analysis on HCC number in five animal experiments. Of these five experiments, four showed a significant decrease of HCC number and none showed a significant increase. Although it was unclear how the data were presented in DePeralta’s study [14], we assumed they were presented as mean ± SE. The result didn’t show significant inhibitory effect on HCC tumor number by metformin (SMD-1.05[-2.13,0.03]; n = 5) (Fig 4). We found high heterogeneity (I^2^ = 78%).

### Effects of metformin on HCC incidence

**Fig 5:**
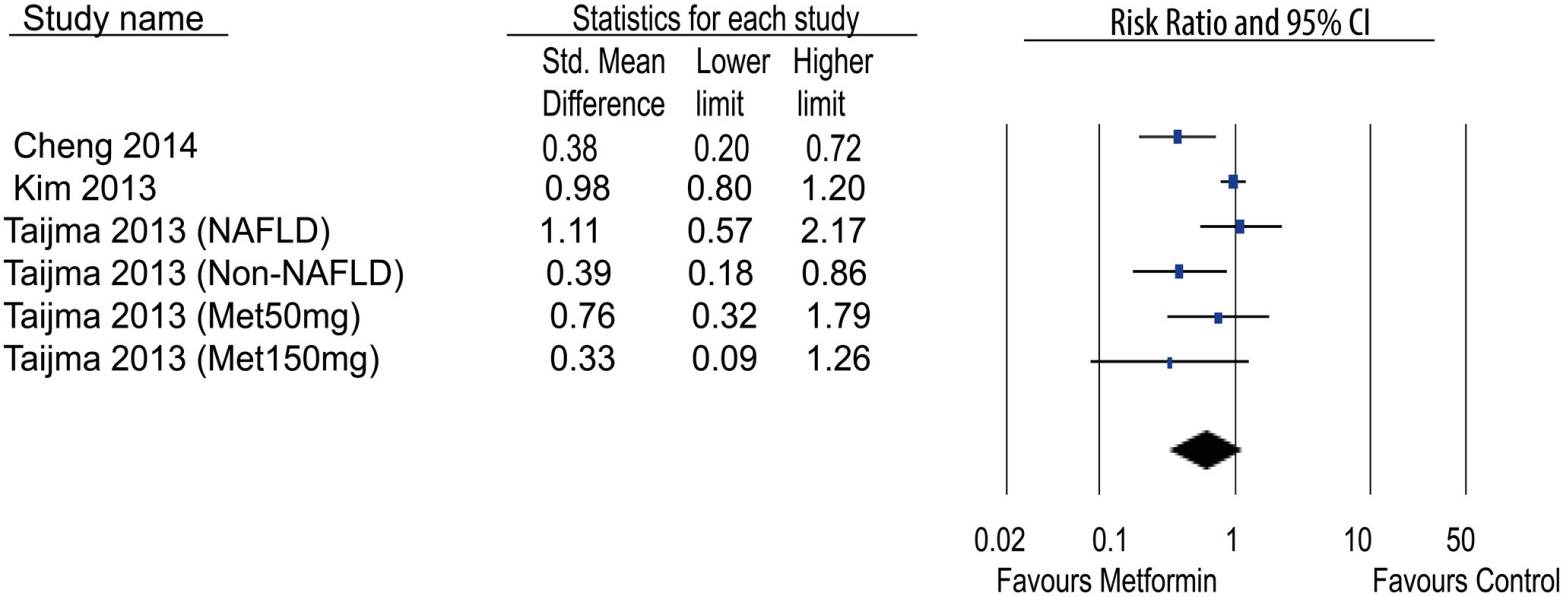
Effects of metformin on tumor incidence in HCC animal models.

The effects of metformin on the incidence of HCC in animal models was evaluated. Only two studies showed a significant decrease of HCC incidence; whereas the others showed trend of decrease but one showed a trend of increase. However, the meta-analysis didn’t show a significant effect of metformin on the incidence of HCC in comparison with non-treatment group (RR 0.62[0.33,1.16]; n = 6) (Fig 5). The heterogeneity was high (I^2^ = 83%).

### Sensitivity analysis

We performed sensitivity analysis to assess the robustness of our results on the effects of metformin on HCC number. In the subgroup analysis of “tumor number”, we assumed that DePeralta[14]presented the data as mean ± SE. For this sensitivity analysis we test the assumption that the data were presented as mean ± SD. With this assumption the meta-analysis showed that metformin could significantly reduce HCC tumor number (SMD-1.21[-2.29, -0.13];n = 5). This result differs from our previous findings in the subgroup analysis. Interpretation of this outcome measure should be done with extreme caution, as current available evidence is still inconclusive.

### Publication bias

**Fig 6:**
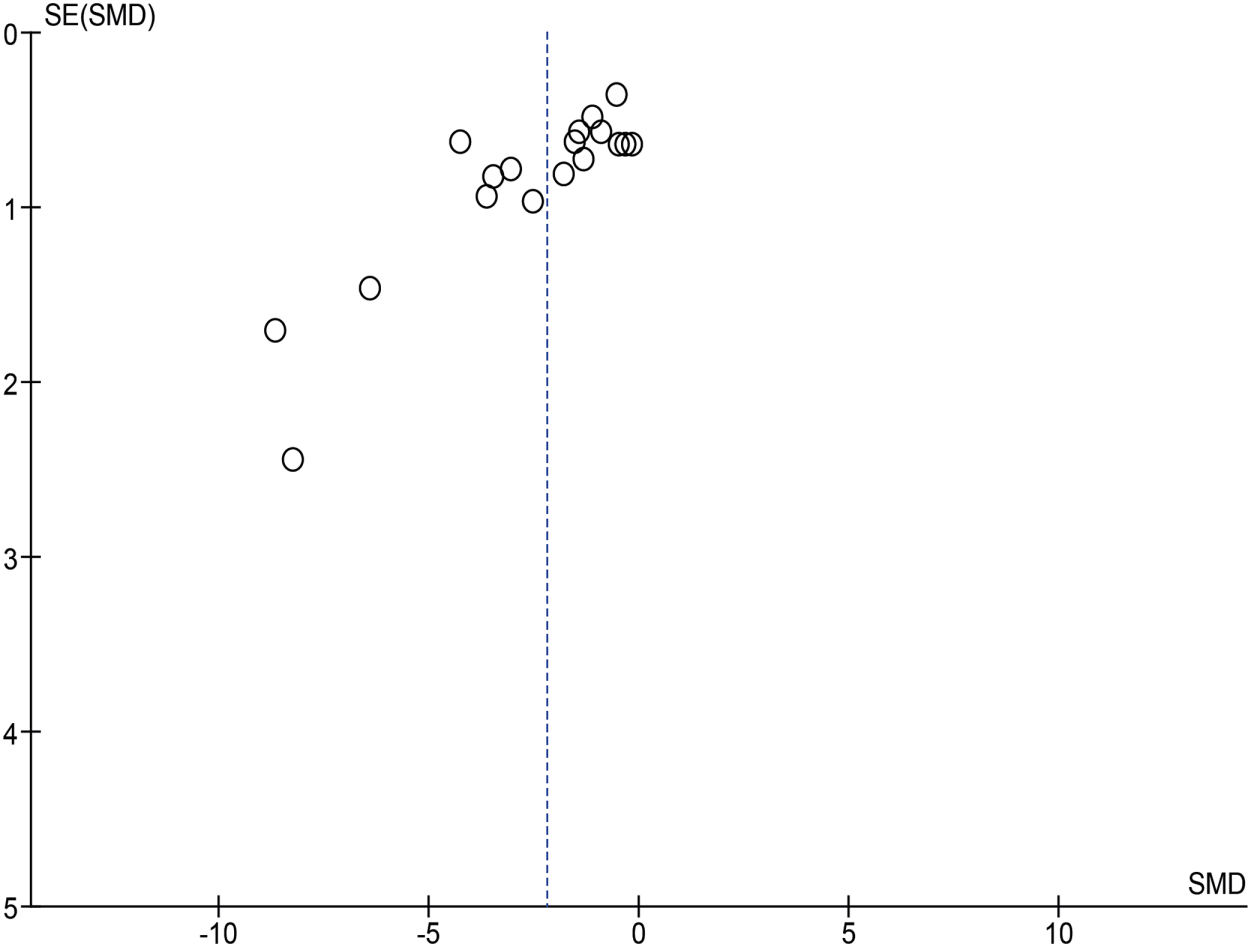
Funnel plot overseeing publication bias of included studies.

Publication bias was assessed for the outcome of overall tumor growth, since the analysis of this outcome included the highest number of studies. On visual inspection of the funnel plot (Fig 6), small studies with no or a negative effect seem to be missing. This asymmetry might indicate the presence of publication bias.

## Discussion

Although there were already several clinical studies evaluating the effects of metformin on HCC risk in human population, all of them were on the chemo-preventive effect by metformin rather than the therapeutic effect and, moreover, the population were all diabetic patients. A meta-analysis of the effects of anti-diabetic medications on the HCC risk, in which both observational and RCT studies were included, has suggested a half reduction in HCC incidence when using metformin treatment [22]. However, our meta-analysis systematically analyzed all relevant animal studies to assess the therapeutic potential of metformin against HCC.

In this comprehensive systematic review and meta-analysis, we analyzed and described the effects of metformin on HCC growth and incidence. The overall analysis of the effect of growth showed that use of metformin was associated with a significant inhibitory effect (SMD = -2.20±0.76) on HCC growth, compared to untreated group. This therapeutic effect remained stable across subgroups of tumor volume, tumor weight and tumor size. It was most pronounced in “tumor volume” measurement while least in “tumor size” measurement. Besides, we performed subgroup analysis under the “tumor growth”. Both xenograft and non-xenograft studies showed significant inhibitory effect of metformin on HCC growth.

### Mechanism underlying metformin anti-HCC

The anti-cancer effect of metformin was speculated to be associated with the activation of adenosine monophosphate-activated protein kinase (AMPK). An important upstream kinase of AMPK is LKB1, an very important tumor suppressor [23,24]. This signaling pathway was also discussed and explored in the included HCC animal trials. The coexistence of AMPK-dependent and AMPK-independent mechanisms for the effects of metformin on cancer was proposed[9,10,12,15]. But we should keep in mind of different study methodologies. For instance, two studies used cell lines *in vitro*[9,15]; one study used tumor harvested from xenograft[10]; whereas one study demonstrated their result based on observing AMPK level in liver[12]. In addition, metformin may also inhibit HCC cell growth by regulating cell-cycle regulatory proteins, such as cyclin D1 and cyclin E [10,14]. Of particular note, c-myc was suggested as a critical mediator in hepatocarcinogenesis [25]. Metformin treatment has been shown to inhibit c-myc expression by up-regulating let-7 family (tumor suppressor) [11]. However, no study among the included animal studies discussed such mechanism underlying specific HCC stage. Although we found one human study relevant to early stage of HCC, no clear mechanistic insight of metformin on early stage HCC was described[26].

### Side effects of metformin in animal models

Being a classic antidiabetic medication, metformin is widely used among patients because of its relatively low cost and high safety profile. 10 out of 13 studies included in this systematic review mentioned tolerability of metformin treatment. These studies consistently showed that metformin didn’t change body weight and serum glucose level of animals. However, it should also be taken into consideration of further investigation on the appropriate therapeutic dosage rang of metformin for anti-cancer treatment. Evaluation of safety with these particular dosages is very necessary, especially when metformin is applied in non-diabetic patients.

### Limitations

By using the risk of bias tool, we found out that reporting is poor and therefore the methodological quality of many studies is unclear. It shows that there is much room for improvement, since many items were shown “unclear” and only a few items were shown low risk only in very few studies. Besides, by visualizing the funnel plot, publication bias seems also to be present, probably due to the missing of studies with no or negative effects. Actually, in total, only 12 studies were included in the meta-analysis. Both the unclear methodological quality and publication bias might lead to under or overestimation of the overall effect size of metformin effect on anti-HCC, which is an additional threat to the robustness of the data especially the ones that are already inconclusive. What’s more, high heterogeneity was also seen among the studies, which is common in animal studies, although we tried to explore the potential factors contributing the heterogeneity. All of these potential limitations might influence us to draw concrete conclusions on the anti-HCC effect of metformin.

Besides, there were some methodological issues which might influence the translation of animal results to human trials. Firstly, the literature is unclear about which animal method is most representative for patient HCC. We found various HCC methods used in these studies: xenograft, DEN-induction, transgenic and dietary models. Secondly, there are two different administration routes (oral or i.p.) of metformin in the studies. However, metformin is usually an orally administrated drug in clinic, raising the question whether administration method could also affect the effect of metformin on tumor.

HCC invasion and metastasis are crucial factors related to poor prognosis. However, none of the animal studies described if metformin had potential effect on HCC metastasis, except one study indirectly mentioned correlation of metformin/p-AMPK with distant metastasis in human HCC cohort study [19].

Another limitation is that these animal studies did not study the HCC stage indicated for metformin. HCC stage could be an important factor for the therapeutic efficacy of metformin and has implication for selecting appropriate candidates for metformin treatment. Therefore, it’s very necessary to take the stage of HCC into account in the experimental design. As for xenograft animal model, which were used in most of the studies, however, it’s hard to define the cancer stage which didn’t discuss in the studies. In addition, although it’s possible to identify tumor stage in spontaneous animal tumor model, the authors did not report any information about HCC stage in their animal study[12,17].

### Implications for practice

Based on the results of this meta-analysis, metformin could potentially have a therapeutic effect on HCC. Besides, the maximal dose of metformin used in all the included animal studies were consistent with human therapeutic dose in diabetics according to the calculation [10]. This furthermore supported the reliable and applicable results from animal models. Although several clinical studies reported that metformin could negatively modify the risk of HCC in diabetic patients [4–6], there are not yet any clinical trial investigating the therapeutic effect of metformin on HCC. Currently, several ongoing clinical trials (www.clinicaltrials.gov) are evaluating the effects of metformin on different cancers (breast cancer, colorectal cancer, pancreatic cancer, etc.), But not for HCC. Thus, the results of this systematic review provide an important reference for the future preclinical animal trials with high quality to aid the development of metformin for anti-HCC treatment in clinical trials. Considering the dominant risk factor for HCC in many western countries in particular United States--obesity and metabolic syndrome, metformin would be an appropriate choice for such a high-risk of population of HCC.

## Conclusion

In summary, this systematic review of animal studies suggests that metformin potentially has a direct inhibitory effect on HCC growth, although the effects on tumor number and incidence are inconclusive. It supports the clinical observation that metformin is associated with lower risk of HCC in diabetic patients. Although these animal studies have some intrinsic limitations, these results do provide an important reference for future high-quality preclinical animal trials and potential clinical development.

## Supporting Information

S1 Table Search Strategy. (DOCX)

S2 Table PRISMA Checklist for the Systematic Review and Meta-analysis to Estimate the anti-HCC effect of metformin in animal studies. (DOC)
