# Supplementary material for: Anti-Tumor Effects of Metformin in Animal Models of Hepatocellular Carcinoma: A Systematic Review and Meta-Analysis
Source: PLoS One. 2015 Jun 1;10(6):e0127967. doi: 10.1371/journal.pone.0127967 (PMC4451077; doi:10.1371/journal.pone.0127967)
Supplement: S1 Table — (DOCX) [file pone.0127967.s001.docx]

| embase.com | 133 |
| --- | --- |
| Component 1: hepatocellular carcinoma | ('liver cell carcinoma'/de OR 'hepatocellular carcinoma cell line'/exp OR 'hepatoma cell'/de OR (((liver OR hepat*) NEAR/6 (carcino* )) OR hepatoma* OR (hepatocell* NEAR/3 cancer*) OR hepatocarcinom* OR hepatoma OR 'AH 109a' OR AH109a OR 'AH 130' OR AH130 OR 'AH 272' OR AH272 OR 'AH 66' OR AH66 OR HepG2 OR 'Hep G2' OR hcc):ab,ti) |
| Component 2: metformin | AND (metformin/de OR (metformin* OR metphormin* OR methformin* OR metaformin* OR dimethylbiguanide OR 'dimethyl biguanide' OR dimethyldiguanide OR dimethylguanylguanidine OR apophage OR aron OR benofomin OR dabex OR denkaform OR deson OR dextin OR diabetase OR diabetformin OR diabetmin OR diabetosan OR diabex OR diafat OR diaformin OR diaformina OR diametin OR diamin OR diformin OR dimefor OR dimethylbiguanide OR dimethyldiguanide OR dmgg OR dybis OR eraphage OR espa-formin OR 'euform retard' OR fluamine OR flumamine OR fornidd OR fortamet OR glafornil OR glibudon OR glifage OR gliguanid OR glucaminol OR glucofage OR glucofago OR glucoform OR glucoformin OR glucohexal OR glucoless OR glucomet OR glucomin OR glucomine OR gluconil OR glucophage OR glucotika OR gludepatic OR glufor OR gluformin OR glumeformin OR glumet OR glumetza OR glupa OR glustress OR glyciphage OR glycomet OR glycon OR glycoran OR glyformin OR glymet OR haurymellin OR hipoglucin OR i-max OR islotin OR juformin OR 'la 6023' OR la6023 OR maformin OR meglucon OR meguan OR melbin OR melformin OR mellittin OR mescorit OR metaformin OR metfogamma OR metforal OR metformin* OR methformin OR metiguanide OR metomin OR metphormin OR miformin OR neoform OR nndg OR reglus-500 OR riomet OR siamformet OR siofor OR thiabet OR vimetrol OR walaphage):ab,ti) |
| Component 3: animal | NOT ([humans]/lim NOT [animals]/lim) |
| Medline (OvidSP) | **82** |
| Component 1: hepatocellular carcinoma | ("Carcinoma, Hepatocellular"/ OR (((liver OR hepat*) ADJ6 (carcino* )) OR hepatoma* OR (hepatocell* ADJ3 cancer*) OR hepatocarcinom* OR "AH 109a" OR AH109a OR "AH 130" OR AH130 OR "AH 272" OR AH272 OR "AH 66" OR AH66 OR HepG2 OR "Hep G2" OR hcc).ab,ti.) |
| Component 2: metformin | AND (metformin/ OR (metformin* OR metphormin* OR methformin* OR metaformin* OR dimethylbiguanide OR "dimethyl biguanide" OR dimethyldiguanide OR dimethylguanylguanidine OR apophage OR aron OR benofomin OR dabex OR denkaform OR deson OR dextin OR diabetase OR diabetformin OR diabetmin OR diabetosan OR diabex OR diafat OR diaformin OR diaformina OR diametin OR diamin OR diformin OR dimefor OR dimethylbiguanide OR dimethyldiguanide OR dmgg OR dybis OR eraphage OR espa-formin OR "euform retard" OR fluamine OR flumamine OR fornidd OR fortamet OR glafornil OR glibudon OR glifage OR gliguanid OR glucaminol OR glucofage OR glucofago OR glucoform OR glucoformin OR glucohexal OR glucoless OR glucomet OR glucomin OR glucomine OR gluconil OR glucophage OR glucotika OR gludepatic OR glufor OR gluformin OR glumeformin OR glumet OR glumetza OR glupa OR glustress OR glyciphage OR glycomet OR glycon OR glycoran OR glyformin OR glymet OR haurymellin OR hipoglucin OR i-max OR islotin OR juformin OR "la 6023" OR la6023 OR maformin OR meglucon OR meguan OR melbin OR melformin OR mellittin OR mescorit OR metaformin OR metfogamma OR metforal OR metformin* OR methformin OR metiguanide OR metomin OR metphormin OR miformin OR neoform OR nndg OR reglus-500 OR riomet OR siamformet OR siofor OR thiabet OR vimetrol OR walaphage).ab,ti.) |
| Component 3: animal | NOT (humans/ NOT animals/) |
| Web-of-science | **100** |
| Component 1: hepatocellular carcinoma | TS=(((((liver OR hepat*) NEAR/6 (carcino* )) OR hepatoma* OR (hepatocell* NEAR/3 cancer*) OR hepatocarcinom* OR hepatoma OR "AH 109a" OR AH109a OR "AH 130" OR AH130 OR "AH 272" OR AH272 OR "AH 66" OR AH66 OR HepG2 OR "Hep G2" OR hcc)) |
| Component 2: metformin | AND ((metformin* OR metphormin* OR methformin* OR metaformin* OR dimethylbiguanide OR "dimethyl biguanide" OR dimethyldiguanide OR dimethylguanylguanidine OR apophage OR aron OR benofomin OR dabex OR denkaform OR deson OR dextin OR diabetase OR diabetformin OR diabetmin OR diabetosan OR diabex OR diafat OR diaformin OR diaformina OR diametin OR diamin OR diformin OR dimefor OR dimethylbiguanide OR dimethyldiguanide OR dmgg OR dybis OR eraphage OR espa-formin OR "euform retard" OR fluamine OR flumamine OR fornidd OR fortamet OR glafornil OR glibudon OR glifage OR gliguanid OR glucaminol OR glucofage OR glucofago OR glucoform OR glucoformin OR glucohexal OR glucoless OR glucomet OR glucomin OR glucomine OR gluconil OR glucophage OR glucotika OR gludepatic OR glufor OR gluformin OR glumeformin OR glumet OR glumetza OR glupa OR glustress OR glyciphage OR glycomet OR glycon OR glycoran OR glyformin OR glymet OR haurymellin OR hipoglucin OR i-max OR islotin OR juformin OR "la 6023" OR la6023 OR maformin OR meglucon OR meguan OR melbin OR melformin OR mellittin OR mescorit OR metaformin OR metfogamma OR metforal OR metformin* OR methformin OR metiguanide OR metomin OR metphormin OR miformin OR neoform OR nndg OR reglus-500 OR riomet OR siamformet OR siofor OR thiabet OR vimetrol OR walaphage)) |
| Component 3: animal | AND (mice OR mouse OR rat OR rats)) |
| Scopus | **106** |
| Component 1: hepatocellular carcinoma | TITLE-ABS-KEY(((((liver OR hepat*) W/6 (carcino* )) OR hepatoma* OR (hepatocell* W/3 cancer*) OR hepatocarcinom* OR hepatoma OR "AH 109a" OR AH109a OR "AH 130" OR AH130 OR "AH 272" OR AH272 OR "AH 66" OR AH66 OR HepG2 OR "Hep G2" OR hcc)) |
| Component 2: metformin | AND ((metformin* OR metphormin* OR methformin* OR metaformin* OR dimethylbiguanide OR "dimethyl biguanide" OR dimethyldiguanide OR dimethylguanylguanidine OR apophage OR aron OR benofomin OR dabex OR denkaform OR deson OR dextin OR diabetase OR diabetformin OR diabetmin OR diabetosan OR diabex OR diafat OR diaformin OR diaformina OR diametin OR diamin OR diformin OR dimefor OR dimethylbiguanide OR dimethyldiguanide OR dmgg OR dybis OR eraphage OR espa-formin OR "euform retard" OR fluamine OR flumamine OR fornidd OR fortamet OR glafornil OR glibudon OR glifage OR gliguanid OR glucaminol OR glucofage OR glucofago OR glucoform OR glucoformin OR glucohexal OR glucoless OR glucomet OR glucomin OR glucomine OR gluconil OR glucophage OR glucotika OR gludepatic OR glufor OR gluformin OR glumeformin OR glumet OR glumetza OR glupa OR glustress OR glyciphage OR glycomet OR glycon OR glycoran OR glyformin OR glymet OR haurymellin OR hipoglucin OR i-max OR islotin OR juformin OR "la 6023" OR la6023 OR maformin OR meglucon OR meguan OR melbin OR melformin OR mellittin OR mescorit OR metaformin OR metfogamma OR metforal OR metformin* OR methformin OR metiguanide OR metomin OR metphormin OR miformin OR neoform OR nndg OR reglus-500 OR riomet OR siamformet OR siofor OR thiabet OR vimetrol OR walaphage)) |
| Component 3: animal | AND (mice OR mouse OR rat OR rats OR animal*)) |
| PubMed Publisher | **2** |
| Component 1: hepatocellular carcinoma | ((((liver OR hepat*[tiab]) AND (carcino*[tiab] )) OR hepatoma*[tiab] OR (hepatocell*[tiab] AND cancer*[tiab]) OR hepatocarcinom*[tiab] OR AH 109a OR AH109a OR AH 130 OR AH130 OR AH 272 OR AH272 OR AH 66 OR AH66 OR HepG2 OR Hep G2 OR hcc)) |
| Component 2: metformin | AND ((metformin*[tiab] OR metphormin*[tiab] OR methformin*[tiab] OR metaformin*[tiab] OR dimethylbiguanide OR dimethyl biguanide OR dimethyldiguanide OR dimethylguanylguanidine OR apophage OR aron OR benofomin OR dabex OR denkaform OR deson OR dextin OR diabetase OR diabetformin OR diabetmin OR diabetosan OR diabex OR diafat OR diaformin OR diaformina OR diametin OR diamin OR diformin OR dimefor OR dimethylbiguanide OR dimethyldiguanide OR dmgg OR dybis OR eraphage OR espa-formin OR euform retard OR fluamine OR flumamine OR fornidd OR fortamet OR glafornil OR glibudon OR glifage OR gliguanid OR glucaminol OR glucofage OR glucofago OR glucoform OR glucoformin OR glucohexal OR glucoless OR glucomet OR glucomin OR glucomine OR gluconil OR glucophage OR glucotika OR gludepatic OR glufor OR gluformin OR glumeformin OR glumet OR glumetza OR glupa OR glustress OR glyciphage OR glycomet OR glycon OR glycoran OR glyformin OR glymet OR haurymellin OR hipoglucin OR i-max OR islotin OR juformin OR la 6023 OR la6023 OR maformin OR meglucon OR meguan OR melbin OR melformin OR mellittin OR mescorit OR metaformin OR metfogamma OR metforal OR metformin*[tiab] OR methformin OR metiguanide OR metomin OR metphormin OR miformin OR neoform OR nndg OR reglus-500 OR riomet OR siamformet OR siofor OR thiabet OR vimetrol OR walaphage)) |
| Component 3: animal | AND (mice OR mouse OR rat OR rats OR animal*) AND publisher[sb] |
| Google Scholar | **150** |
| Component 1: hepatocellular carcinoma | "liver cell carcinoma"\|"hepatocellular carcinoma\|cancer"\|hepatoma\|hepatocarcinoma |
| Component 2: metformin | Metformin |
| Component 3: animal | mice\|mouse\|rat\|rats\|animal\|animals |
